# Predicting gestational diabetes before conception for personalized interpregnancy weight management

**DOI:** 10.1038/s41598-025-30028-y

**Published:** 2025-11-27

**Authors:** Sho Tano, Tomomi Kotani, Tatsuo Inamura, Kazuya Fuma, Seiko Matsuo, Masato Yoshihara, Kenji Imai, Masataka Nomoto, Yoshinori Moriyama, Shigeru Yoshida, Mamoru Yamashita, Yasuyuki Kishigami, Hidenori Oguchi, Takafumi Ushida, Hiroaki Kajiyama

**Affiliations:** 1 Department of Obstetrics and Gynecology, Nagoya University Graduate School of Medicine, Showa-ku Tsurumai 65, Nagoya, Aichi 466-8560 Japan; 2 Department of Obstetrics, Perinatal Medical Center, TOYOTA Memorial Hospital, Toyota, Aichi Japan; 3 Department of Obstetrics & Gynecology, Hamamatsu University School of Medicine, Hamamatsu, Shizuoka 431-3192 Japan; 4 Department of Obstetrics and Gynecology, Ogaki Municipal Hospital, Ogaki, Gifu Japan; 5 Department of Obstetrics and Gynecology, Fujita Health University School of Medicine, Toyoake, Aichi Japan; 6 Kishokai Medical Corporation, Nagoya, Aichi Japan

**Keywords:** Inter-conception care, Obesity, Overweight, Pre-conception care, Diseases, Endocrinology, Health care, Medical research, Risk factors

## Abstract

The growing recognition of the importance of interpregnancy care to reduce gestational diabetes mellitus (GDM) risk underscores the importance of effective preventive strategies. However, developing effective systems is still challenging. We aimed to bridge this gap by developing a weight management specific prediction model. This study retrospectively analyzed the data of women who underwent two childbirths across 15 medical facilities, including both primary and tertiary facilities. A derivation cohort was constructed using data from 2009 to 2019 (n = 1,640). Data between 2020 and 2024 was used to construct a separate temporal-validation cohort (n = 293). Using the data from another tertiary center between 2017 and 2023, the geographical-validation cohort was constructed (n = 339). A prediction model for GDM development in the second pregnancy was developed by applying logistic regression analysis using 5 key clinical information. GDM in the second pregnancy occurred in 9.5% (156 of 1,640, derivation), 16.7% (49 of 293, temporal-validation), and 7.7% (26 of 339, geographical-validation). The prediction model demonstrated consistent discrimination across cohorts, with c-statistics of 0.75, 0.80, and 0.79, respectively. Precision–recall analyses, accounting for the low prevalence of GDM, further confirmed performance well above the baseline (0.095 in the derivation cohort), with AUC-PRs of 0.43, 0.47, and 0.41 for the three cohorts. Calibration showed alignment with slopes of 1.04, 0.87, and 0.59 for each cohort. This simple and accurate model supports personalized weight management goals, offering a practical tool to reduce GDM risk in future pregnancies through inter-pregnancy weight management.

**Supplementary Information:**

The online version contains supplementary material available at 10.1038/s41598-025-30028-y.

## Introduction

Gestational diabetes mellitus (GDM) affects 7–28% of pregnancies worldwide and is a significant contributor to neonatal mortality^1–3^. With a significant increase in the reported incidences of GDM, preventive strategies are gaining importance. Preventive strategies for GDM are divided into two phases: pre-conception and post-conception (during pregnancy)^4–7^. Managing gestational weight gain (weight gain during pregnancy) is a critical preventive measure after conception^4,5^. However, maintaining appropriate pre-pregnancy body weight is essential for reducing GDM risk^6–11^

Inter-pregnancy care (IPC) has been advocated as a practical pre-conception strategy for preventing GDM^8–10,12^. Additionally, IPC interventions are gaining attention for their potential to extend the healthy life expectancy of women^10,13^. The general guidance for weight management between pregnancies (inter-pregnancy weight management), as part of IPC, recommends maintaining a body mass index (BMI) between 18.5 and 25.0 kg/m^2^ to reduce GDM risk in the subsequent pregnancy^8,10,14^. However, this BMI target can be challenging for women with severe obesity, who may benefit most from weight management.

In preventive medicine, two principal strategies are commonly distinguished: the population approach and the high-risk approach^15,16^. The population approach seeks to reduce risk at a community level by shifting the overall distribution of risk, whereas the high-risk approach focuses on individuals at greatest risk through tailored interventions. In our previous work^7^, we showed that weight gain of up to +0.6 kg/m^2^/year during the IPC period was not associated with increased GDM risk, providing a benchmark for population approach. By contrast, the high-risk approach requires reliable prediction models to identify women most likely to benefit from intensive intervention. Individualized weight targets derived from such models could enhance preventive efficiency by concentrating support on high-risk women while avoiding unnecessary burden on those at lower risk.

The advantage of employing such prediction models for setting weight management targets lies not only in their capacity to tailor goals to individual risk profiles, but also in facilitating shared decision-making between patients and healthcare providers. This enables a balanced consideration of the anticipated burden of weight control efforts against the potential reduction in future disease risk. This approach would operationalize the proposed strategy as follows: the process begins with identifying risk factors from the prior pregnancy (Step 1: risk assessment), proceeds to planning the interpregnancy interval (Step 2: family planning), and culminates in the application of the prediction model to establish personalized weight management goals based on the estimated reduction in the risk of GDM onset in subsequent pregnancies (Step 3: goal setting)^17^.

Historically, GDM prediction models have primarily focused on post-conception measures^18–21^. Remarkably, none concentrated on pre-conception measures, including inter-pregnancy weight management. Therefore, our approach aims to enable risk stratification before pregnancy to inform IPC.

## Methods

### Cohorts

A dataset from our previous study was used as the derivation cohort^7^. This dataset comprised the electronic medical records of women aged 15 years or older with singleton pregnancies who had both an index pregnancy and a subsequent pregnancy during the study period at two tertiary care facilities (Nagoya University Hospital and TOYOTA Memorial Hospital in Aichi, Japan) and 12 private obstetric clinics (Kishokai Medical Corporation in Aichi and Gifu, Japan) between 2009 and 2019. The first and second pregnancies during this period were designated as index and subsequent pregnancies, respectively. Notably, this inclusion criterion reflects the availability of two consecutive deliveries within the study period and should not be confused with parity; women whose index pregnancy was their first delivery (primiparous) as well as those with prior deliveries (multiparous) were both included.

**Fig. 1:**
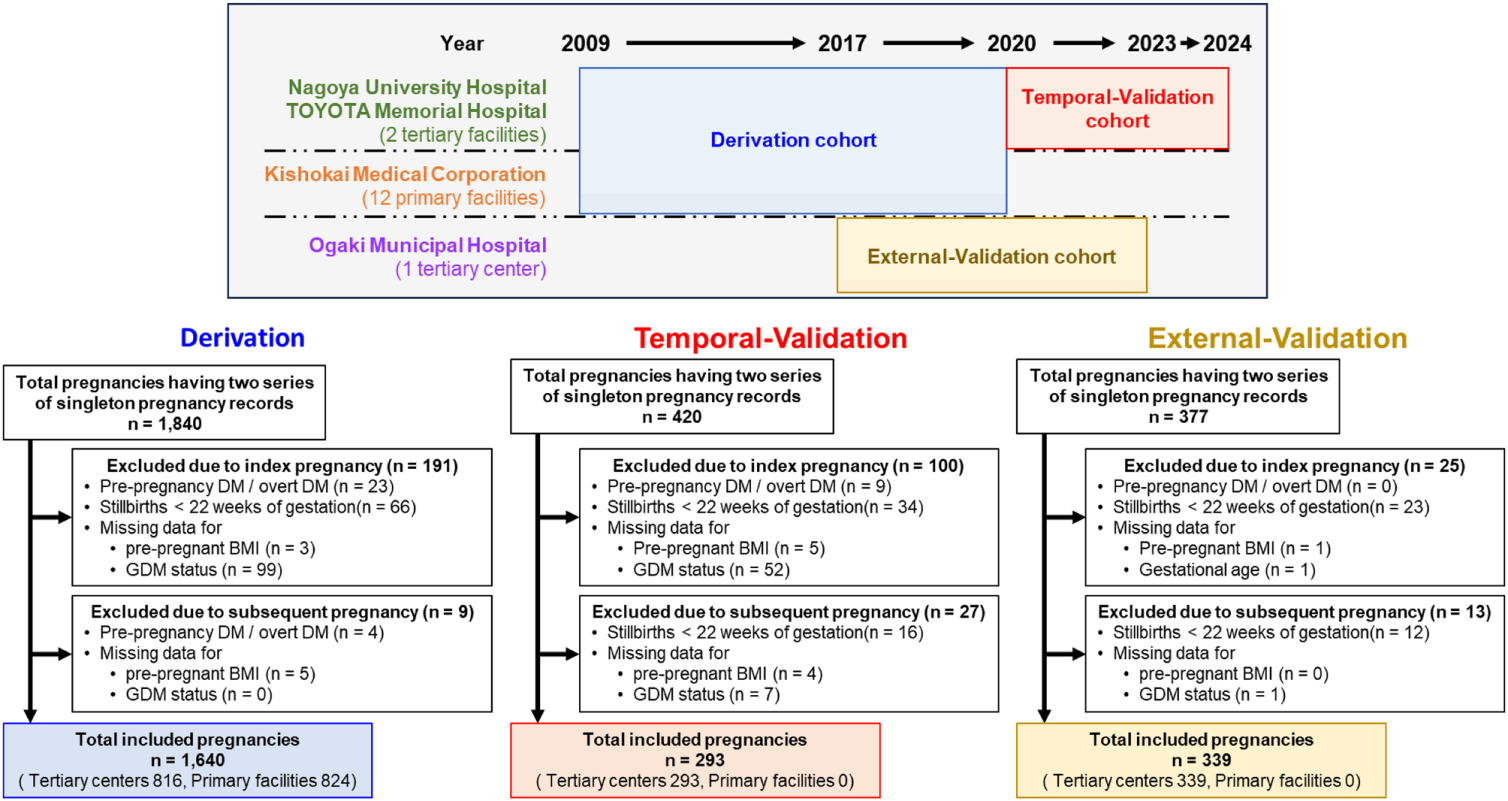
Flowchart Showing the Composition of the Derivation, Temporal-Validation, and Geographical-Validation Cohorts. This figure illustrates the selection process for the study cohort. The derivation cohort included participants from 12 primary care facilities and 2 tertiary hospitals, with subsequent pregnancies resulting in deliveries between 2009 and 2019, as indicated by a blue box. By contrast, the temporal validation cohort comprised participants exclusively from two tertiary hospitals between 2020 and 2024, as indicated by the red boxes. The geographical validation cohort comprised participants from a different tertiary hospital between 2017 and 2023, as indicated by the gold box. DM, diabetes mellitus; BMI, body mass index; GDM, gestational diabetes mellitus.

Women were excluded if they had pre-pregnancy DM, overt DM, multiple pregnancies, stillbirths before 22 weeks of gestation, or missing data on pre-pregnancy BMI and GDM status (Fig. 1). For subsequent pregnancies, the same exclusion criteria were applied to ensure comparability across cohorts.

Using identical inclusion and exclusion criteria, data from two tertiary centers were retrospectively collected for the period between January 2020 and June 2024. Women with singleton pregnancies who delivered a subsequent pregnancy during this timeframe were included in the temporal validation cohort. Importantly, the individuals in the derivation and temporal validation cohorts were mutually exclusive, ensuring independence between the two groups. Additionally, a geographical validation cohort was established using data from a separate tertiary center, Ogaki Municipal Hospital in Gifu Prefecture, covering the period from January 2017 to December 2023.

This study was approved by the ethics board of Nagoya University Hospital (approval number: 2015–0415) and was conducted in accordance with the Declaration of Helsinki. We adhered to the Transparent Reporting of a Multivariable Prediction Model for Individual Prognosis or Diagnosis (TRIPOD) guidelines. The requirement for informed consent was waived due to the retrospective nature of the study.

### Outcomes

The primary outcome was the occurrence of GDM during subsequent pregnancies (GDM^sub^). To ensure consistency across the study period (2009–2024), two obstetric specialists (ST and MN) reviewed all medical records and reassessed GDM status uniformly according to the current criteria (75-g OGTT: FPG ≥ 92 mg/dL, 1-h ≥ 180 mg/dL, 2-h ≥ 153 mg/dL)^22^. GDM was diagnosed by using the two-step approach of the Japan Society of Obstetrics and Gynecology^22^. Initially, a random blood glucose test or a non-fasting 50-g blood glucose challenge test was administered with cutoff values of 100 mg/dL or 140 mg/dL, respectively. In this study, the derivation and temporal validation cohorts adopted the 50-g glucose challenge test as the initial screening, whereas the geographical validation cohort employed random blood glucose screening. Importantly, all women underwent initial screening in their both index and subsequent pregnancy, regardless of prior GDM history. For those with positive screening results, a 75 g oral glucose tolerance test (75 g OGTT) was conducted. GDM was diagnosed if any of the following plasma glucose values were met: a fasting plasma glucose level (FPG) of ≥92 mg/dL, a 1-hour plasma glucose level (1h-PG) of ≥180 mg/dL, or a 2-hour plasma glucose level (2h-PG) of ≥153 mg/dL. All the 15 collaborating institutions used the same diagnostic protocols.

### Definitions of variables

Overt DM was defined as a pre-pregnancy DM diagnosis, HbA1c ≥6.5% (48 mmol/mol), or FPG ≥126 mg/dL during pregnancy^22^. For both the index and subsequent pregnancies, maternal pre-pregnancy BMI was calculated as weight in kilograms divided by height in meters squared (kg/m^2^). Pre-pregnancy body weight was obtained by maternal self-report during routine clinical practice, and height was measured at the first prenatal visit. ΔBMI was defined as the change in pre-pregnancy BMI from the index pregnancy to the subsequent pregnancy. The pregnancy interval was calculated from the expected date of delivery (EDD) of the index pregnancy to that of the subsequent pregnancy, with the EDD determined based on the last menstrual period or crown-rump length measurements during routine practice. Annual BMI change (ABc), which measures the yearly rate of BMI variation between the index and subsequent pregnancies, was calculated as ΔBMI divided by the pregnancy interval (kg/m^2^/year)^7,23,24^.

### Construction of the prediction model

Based on our previous study on HDP prediction, we first considered whether the same minimal set of covariates: maternal age, pre-pregnancy BMI, GDM in index pregnancy [Age^ind^, BMI^ind^, and GDM^ind^, respectively], pregnancy interval (Pi), and ABc^7,18,19,25^. To verify this, we compared prediction models under five different covariate selection scenarios (Figure S1). As described in the Results, calibration analyses indicated that this minimal covariate set (Model 2) achieved adequate predictive accuracy while ensuring parsimony. Prediction models were developed using logistic regression model.

$$Predicted\ probability=\frac{1}{1+{e}^{-Y}}Y=0.060\times {Age}^{ind} + 0.100\times {BMI}^{ind} + 3.143\times {GDM}^{ind} + 0.072\times Pi + 0.450\times ABc - 6.883$$

### Model evaluation

The model performance was evaluated across all three cohorts with respect to discrimination, calibration, and clinical usefulness. Discrimination was evaluated by estimating the area under the receiver operating characteristic curve (ROC) and performing a precision-recall (PR) curve analysis^26,27^. Calibration was evaluated by plotting flexible calibration curves based on locally estimated scatterplot smoothing (LOESS)^28^. Clinical usefulness was evaluated using a decision curve analysis, which calculates the net benefit across various threshold probabilities^29^. Decision Curve Analysis (DCA) was conducted to assess the clinical utility of the prediction models by calculating the “Net Benefit” across various risk thresholds^29^. The DCA compares the performance of three approaches: assuming all patients were at high risk, assuming no patients were at high risk, and using a prediction model.

### Statistical analysis

Differences in characteristics among the three cohorts were statistically analyzed using the chi-square test for categorical variables. Dunnett’s post-hoc one-way analysis of variance (ANOVA) was used to identify significant differences between the derivation and validation cohorts. Statistical significance was set at a two-tailed *p*-value <0.05. Statistical analyses, including model development and performance evaluation, were conducted using R, version 4.1.3 (https://cran.r-project.org/).

## Results

### Baseline characteristics and outcomes of derivation and validation cohorts

The derivation cohort consisted of 1,640 individuals, the temporal validation cohort included 293 individuals, and the geographical validation cohort included 339 individuals (Table 1). There was no missing data in the variables used for analysis, as all cohorts were constructed based on complete-case datasets, and the cohorts were mutually exclusive. In the derivation, temporal validation, and geographical validation cohorts, 9.5% (n=156 1,640), 16.7% (n=49 293), and 7.7% (n=26 339) of individuals developed GDM^sub^, respectively (*p*<0.001).

**Table 1:** Comparison of the derivation and validation cohorts.

	Derivation cohort (n = 1,640)	Temporal-validation cohort (n = 293)	Geographical -validation cohort (n=339)	*p*-value
*Index pregnancy*				
Age, years	30.6 ± 4.8	30.5 ± 4.9	29.2 ± 5.1*	<0.001
Pre-pregnancy BMI, kg/m^2^	20.9 ± 3.4	22.3 ± 4.1*	22.3 ± 4.7*	<0.001
Primiparity	1,302 (79.4)	207 (70.6)	247 (72.9)	<0.001^§^
GDM	70 (4.3)	35 (11.9)	15 (4.4)	<0.001^§^
GA at delivery, weeks	39.1 ± 2.1	39.4 ± 2.8*	38.3 ± 3.0*	<0.001
Birthweight, g	2,966 ± 507	2,823 ± 653*	2,847 ± 633*	<0.001
Macrosomia	14 (0.9)	6 (2.0)	7 (2.1)	0.060
*Interpregnancy period*				
Pregnancy interval, years	2.3 ± 0.9	3.5 ± 2.3*	2.5 ± 1.1*	<0.001
Annual BMI change, kg/m^2^/year	0.21 ± 0.80	0.30 ± 0.83	0.25 ± 1.04	0.232
*Subsequent pregnancy*				
GDM	156 (9.5)	49 (16.7)	26 (7.7)	<0.001^§^
GA at delivery, weeks	39.0 ± 1.6	38.6 ± 2.3*	38.2 ± 2.6*	<0.001
Birthweight, g	3,041 ± 436	2,987 ± 564	2,933 ± 547*	<0.001
Macrosomia	19 (1.2)	5 (1.7)	4 (1.2)	0.733

Data are presented as mean ± standard deviation or n (%).

BMI, body mass index; GDM, gestational diabetes mellitus; GA, gestational age.

^*^Statistically significant compared to the derivation cohort by one-way ANOVA with post-hoc Dunnett’s t-test.

^§^Statistically significant by chi-square test.

In terms of index pregnancy parameters, there were no clinically significant differences in Age among the three groups (30.4 ± 4.8, 30.5 ± 4.9, and 29.2 ± 5.1 years), while it was statistically significant (*p*<0.001). The temporal- and geographical-validation cohorts had slightly higher BMI (20.9 ± 3.4 vs. 22.3 ± 4.1 and 22.3 ± 4.7 kg/m^2^, *p*<0.001) comparing to the derivation cohort. Significant differences were observed in primiparity (79.4% vs. 70.6% vs. 72.9%, *p*<0.001) and GDM incidence (4.3% vs. 11.9% vs. 4.4%, *p*<0.001) between the cohorts.

Regarding interpregnancy parameters, the both validation cohorts exhibited longer pregnancy intervals (2.3 ± 0.9 vs. 3.5 ± 2.3 and 2.5 ± 1.1 years). There was no statistical difference in ABc (0.21 ± 0.80 vs. 0.30 ± 0.83 and 0.25 ± 1.04 kg/m^2^/year).

Furthermore, as shown in Table S1, within the derivation dataset, comparison between the complete-case group used for model construction (n = 1,640) and the excluded individuals with missing data (n = 107) revealed that the missing-data group was slightly younger (29.1 ± 4.9 vs. 30.6 ± 4.8 years, p=0.001) and had shorter interpregnancy intervals (1.8 ± 0.4 vs. 2.3 ± 0.9 years, p<0.001). Although these differences reached statistical significance, their magnitude was small and unlikely to be clinically meaningful. Other maternal and neonatal characteristics, including the subsequent incidence of GDM, were comparable between the two groups.

### Prediction model evaluation

**Fig. 2:**
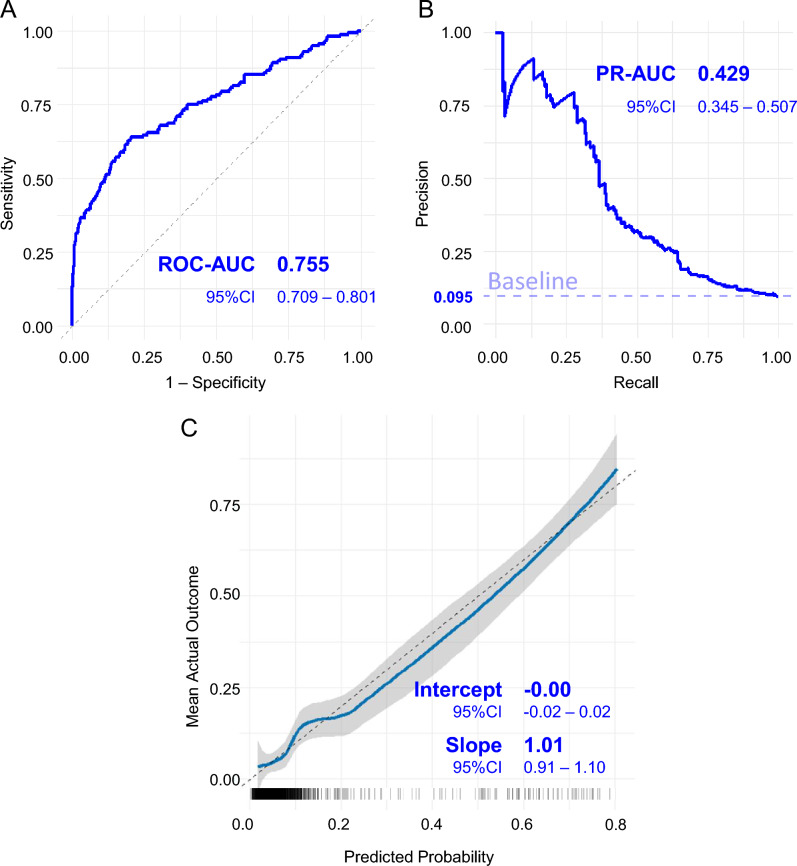
Model Performance for Predicting GDM Risk in the Derivation Cohort. **A**: Receiver Operating Characteristic (ROC) Curves for Prediction Model. This plot displays the ROC curve. The x-axis shows the false-positive rate, whereas the y-axis represents the true-positive rate.** B**: Precision-Recall Curve for Prediction Model. Precision-recall curve illustrates the relationship between recall (x-axis) and precision (y-axis). The dashed horizontal line indicates the baseline precision, corresponding to the prevalence of GDM in the cohort (0.095). This baseline represents the expected precision of random classification, providing a reference against which the model’s performance can be compared. **C**: Calibration Curves for Logistic Regression Model. The plot depicts the agreement between the predicted probability (x-axis) and observed outcomes (y-axis). The shaded areas indicate 95% confidence intervals.

Fig. 2A shows the ROC curve, with an AUC of 0.755 (95% CI: 0.709–0.801), indicating good overall discrimination. Fig. 2B presents the PR curve, reconstructed with the corrected specification and including the prevalence baseline (0.095) as a reference. The AUC-PR was 0.429 (95% CI: 0.345–0.507), confirming that the model outperformed random classification, particularly in the lower recall region where precision remained higher than the baseline. Given the study objective of identifying a prediction model that closely reflects the actual risk of GDM^sub^, calibration analysis was emphasized in the model selection process (Fig. 2C). The model demonstrated favorable calibration, with an intercept of –0.00 (95% CI: –0.02 to 0.02) and a slope of 1.01 (95% CI: 0.91–1.10), indicating close agreement between predicted and observed risks.

Furthermore, calibration analyses across five different variable selection scenarios are presented in Figure S1. All models demonstrated acceptable calibration, with intercepts close to 0 and slopes close to 1. Among them, Model 2, corresponding to the predefined minimal covariate set described in the Methods, was adopted. This approach confirmed that adequate predictive accuracy could be obtained with only a small number of clinically relevant covariates, thereby supporting the goal of developing a practical and parsimonious prediction model.

### Validation

**Fig. 3:**
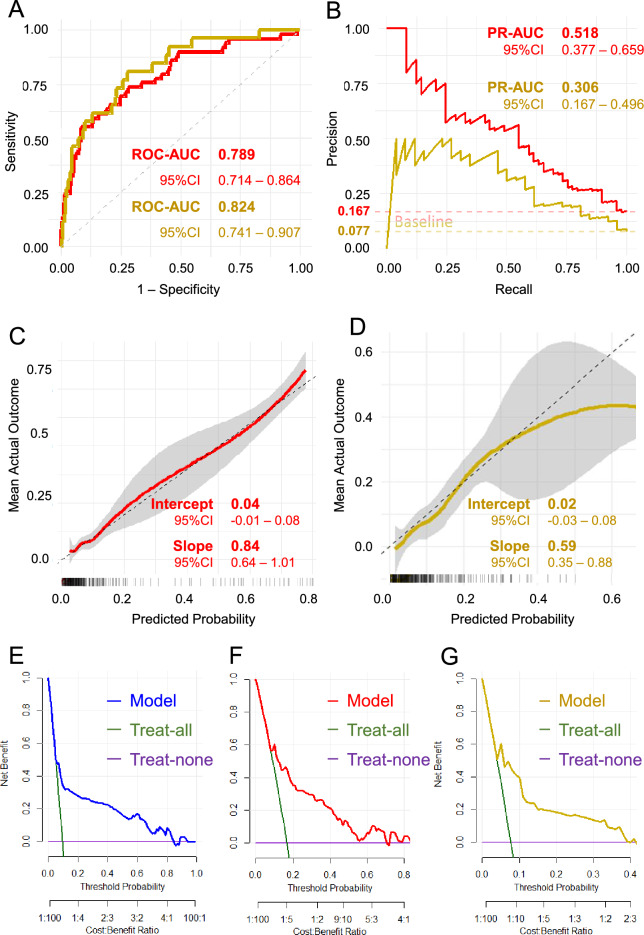
Model Performance Evaluation in Temporal and Geographical Validation Cohorts. **A**: Receiver Operating Characteristic (ROC) Curves. The ROC curves for the temporal validation cohort (red) and geographical validation cohort (gold) are shown, depicting the relationship between the false-positive rate (x-axis) and true-positive rate (y-axis). **B**: Precision-Recall Curves. This panel displays the precision-recall curves for the temporal validation cohort (red) and the geographical validation cohort (gold), with recall on the x-axis and precision on the y-axis. The dashed horizontal lines indicate the baseline precision, corresponding to the prevalence of GDM in each cohort (0.167 for temporal validation and 0.077 for geographical validation). These baselines represent the expected precision of random classification, serving as reference lines for interpreting model performance. **C-D**: Calibration Curves. Calibration analysis for the temporal validation cohort (C, red) and geographical validation cohort (D, gold) cohorts showing agreement between predicted probability (x-axis) and observed outcomes (y-axis). The shaded areas represent 95% confidence intervals. **E-G**: Decision Curve Analysis (DCA).The DCA results compare the net benefit of using the prediction model (Derivation in E [blue], Temporal-validation in F [red], and Geographical-validation in G [gold]), assuming all patients are at high risk (green), or assuming no patients are at high risk (purple) across a range of threshold probabilities (x-axis).

The results shown in Fig. 3 further validate the performance of the prediction model across the temporal- and geographical-validation cohorts. Figure 3A and B present the ROC and precision–recall curves, respectively, for both cohorts. The temporal-validation cohort (red) showed an AUC of 0.789 (95% CI: 0.714–0.864) for ROC analysis, whereas the geographical-validation cohort (gold) had an AUC of 0.824 (95% CI: 0.741–0.907), demonstrating similarly strong discrimination performance. In the Precision-Recall analysis (Fig. 3B), the temporal-validation cohort showed an AUC-PR of 0.518 (95% CI: 0.377–0.659) compared with its baseline of 0.167, while the geographical-validation cohort achieved an AUC-PR of 0.306 (95% CI: 0.167–0.496) against a baseline of 0.077. These findings indicate that in both cohorts, model performance exceeded the baseline precision expected from outcome prevalence, particularly within the low-to-mid recall range where precision remained relatively high, with the temporal-validation cohort demonstrating a more pronounced gain over its baseline.

Calibration analysis for both the temporal- (Fig. 3C) and geographical-validation cohorts (Fig. 3D) showed great performance for predicted probabilities especially below 60%. In this range, the predicted probabilities were closely aligned with the actual outcomes, as indicated by the curve remaining near the ideal calibration line with 95% confidence intervals in both cohorts. However, as the predicted probability exceeded 60%, the overestimation trend (i.e., the predicted probability was greater than the actual outcome) became more evident, particularly in the geographical-validation cohort (Fig. 3D). However, Table S2 shows the distribution predicted probabilities across each cohort. The cumulative percentages of the individuals whose estimated probability of <60% were 96.9%, 92.5 %, and 97.6% for the derivation, temporal-validation, geographical-validation, respectively. Furthermore, the DCA results revealed that in all derivation cohorts, the analysis shows that the model provided a higher Net Benefit than assuming that either all or no patients were at risk across a broad range of risk thresholds (Fig. 3E-G).

## Discussion

**Fig. 4:**
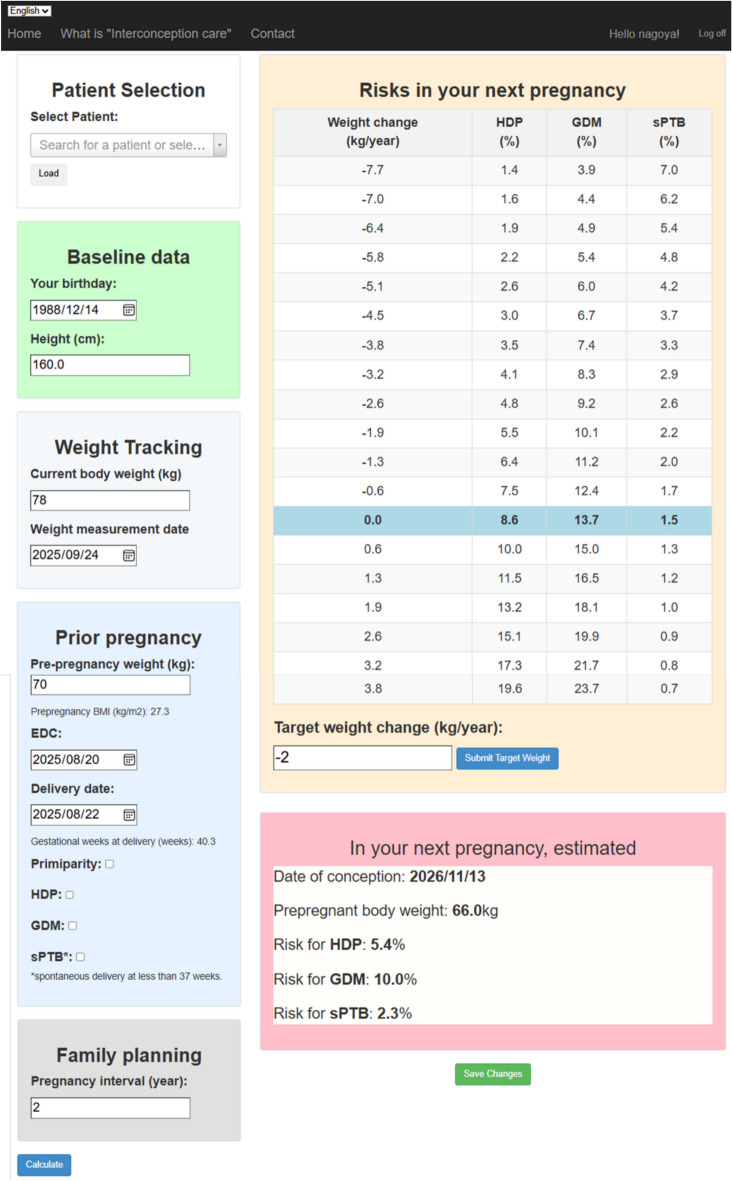
User interface of Web app for interpregnancy weight managementThe interface allows healthcare providers and users to input clinical information following a prior pregnancy and visualize individualized GDM risk estimates based on projected interpregnancy weight change. The tool facilitates shared decision-making by supporting goal setting and lifestyle planning within the interpregnancy interval.

We successfully developed and validated a logistic regression-based prediction model for interpregnancy weight management, which demonstrated high accuracy in forecasting GDM in subsequent pregnancies. This is the first prediction model to concentrate on pre-conception measures; existing prediction models typically assess the risk of GDM during pregnancy^18,19,21^. Unlike conventional supervised learning assumptions, which expect similar characteristics across derivation and validation cohorts^30^, our prediction model maintained reliability despite differences in cohort characteristics. Remarkably, this highly accurate prediction model comprised a simplified set of five key variables, enhancing its practicality for clinical use. Finally, using this model, a web app was developed for personalized risk estimation (Fig. 4).

A recent systematic review of prediction models for GDM concluded that models based solely on maternal information exhibited a predictive accuracy comparable to those of models based solely on various biomarkers^18,20^. This highlights the significant role of maternal characteristics in predicting GDM, particularly clinical factors such as maternal age, GDM history, and pre-pregnancy BMI. Consequently, the high predictive accuracy achieved in our study using only five clinical data points aligns logically with these insights.

Although the diagnostic thresholds for GDM were consistent across all cohorts, the temporal validation cohort demonstrated a higher prevalence of GDM compared to the derivation cohort. This can be attributed to institutional changes at TOYOTA Memorial Hospital, where high-risk pregnancies were increasingly centralized and lower-risk women were managed at peripheral clinics. Consequently, the temporal validation cohort was enriched with a higher-risk case mix compared with the derivation cohort. Importantly, our model maintained satisfactory discrimination and calibration even under this skewed risk distribution. Taken together, these findings indicate that the model performs reliably across different patient risk distributions, supporting its potential applicability in diverse clinical settings.

In our prediction model, there was a tendency for overestimation in individuals with predicted probabilities exceeding 60%, which corresponded to approximately the top 3.6% in terms of frequency. Previous studies on externally validated prediction models have also shown a tendency to overestimate at higher predicted probabilities^31–34^. Possible factors contributing to this overestimation include limitations in the model’s ability to accurately predict such high probabilities. However, because GDM can only be diagnosed through an OGTT, the limitations associated with the diagnostic process may also play a role. The 14 medical institutions that comprised the derivation cohort and temporal-validation cohort used screening based on the 50-g blood glucose challenge test. In contrast, the institutions included in the geographical-validation cohort employed random blood glucose screening. It has been reported that screening with random blood glucose has lower sensitivity^35^, raising the possibility that the reduced implementation of OGTT due to this method may have contributed to a lower diagnostic rate of GDM. Furthermore, it should be noted that screening strategies for GDM are not standardized. In Japan, current guidelines allow either random blood glucose or a 50-g glucose challenge test for initial screening^22^, and practice varies across institutions. Internationally, approaches also differ considerably between countries and regions^36^. This heterogeneity reflects the lack of a universal diagnostic pathway for GDM. Therefore, validating our model in a cohort where random blood glucose screening was adopted provides additional evidence that, although some attenuation in predictive performance was observed, its utility can still be maintained under different diagnostic protocols.

Our prediction model helps women become aware of their GDM risk just after the index pregnancy (during the inter-pregnancy period) and visualize how they can reduce this risk by adjusting the inter-pregnancy weight change. Using the model’s outputs, healthcare providers and women can establish weight management goals through bidirectional communication, thereby facilitating the implementation of inter-pregnancy weight management. This interactive process allows the integration of individual risk tolerance and commitment to lifestyle modifications, enabling personalized and realistic target settings. Women can consider their past experiences and current environment to determine the degree of attainable or appropriate weight loss, whereas healthcare providers can evaluate whether the goals are reasonable. Ultimately, reducing recurrence of GDM is not only relevant for perinatal outcomes but also for long-term health promotion. Women with prior GDM face markedly increased risks of type 2 diabetes and cardiovascular disease^37^. Preventing GDM may therefore help interrupt the trajectory toward chronic metabolic disease, and lifestyle interventions in this group have been shown to reduce future diabetes incidence^38^.

Furthermore, LR-based prediction models can be replicated using software capable of performing logarithmic calculations. Therefore, the reasonable accuracy obtained using logistic regression analysis in our study is advantageous for clinical applications, indicating the potential for high-accuracy predictions, even in low-resource countries and regions.

The primary strength of this study is its novel focus on pragmatic applications for interpregnancy weight management, which is a crucial aspect of IPC. Second, the developed model is rigid and validated using both temporal and geographical validation cohorts. Third, the prediction model, which sets weight management objectives based on annual BMI changes, ensures consistency and reliability, irrespective of variations in pregnancy intervals. Additionally, the model design, which utilizes only five optimal covariates, enhances user-friendliness and reduces the risk of inaccuracy due to missing data in clinical use.

However, this study has some limitations. First, it was retrospective, and annual BMI changes were not intervention-induced. Further studies are required to determine whether intervention-induced weight reduction reduces the risk of developing GDM in subsequent pregnancies. Accordingly, we plan to conduct a prospective study on weight management as part of IPC using the developed prediction model. Second, self-reported body weight was used to calculate the pre-pregnancy BMI. Nonetheless, as almost all participants weighed themselves during antenatal checkups in the first trimester, the difference between their self-reported and actual weights was likely to be minimal. Third, most of the study population comprised Asians; potential model updating is necessary for settings with different BMI distributions/ethnic composition. Fourth, since the model was developed using only complete cases without missing data imputation, a potential risk of bias cannot be excluded. Although the proportion of missing data was relatively small, this approach may limit the robustness of the model. However, the excluded cases with missing values did not differ clinically from the analyzed derivation cohort, suggesting that the impact of this limitation is likely minimal. Another limitation is that our cohorts included only women who delivered both their index and subsequent pregnancies at the same hospital. Such women may represent a selective group influenced by factors such as stable residential location, established trust in the same medical facility, or limited access to alternative providers. Consequently, women who change residence, transfer care, or diversify their choice of delivery hospital were not captured. These factors may introduce selection bias and limit the generalizability of our findings. Finally, it should be noted that our model was developed after excluding women with multiple pregnancies or miscarriage/stillbirth before 22 weeks of gestation. While this approach was necessary to focus on maternal characteristics and interpregnancy changes, it implies that predictive performance is more uncertain for women who may later experience these outcomes. Because the intended use of the model is preconception counseling, users should be reminded that predictions for women at risk of multiple gestations or pregnancy loss may not be as reliable, and further validation in these populations will be required.

## Conclusion

We developed simple and novel inter-pregnancy weight management-focused prediction model. This model is expected to offer personalized risk estimations and a basis for establishing weight management goals intended to reduce the risk of GDM in women planning their next pregnancy.

## Supplementary Information

Supplementary Information 1.

Supplementary Information 2.

Supplementary Information 3.

## Data Availability

The data that support the findings of this study are available from the corresponding author, Sho Tano, upon reasonable request and permission of ethics board.
