# Supplementary material for: Predicting gestational diabetes before conception for personalized interpregnancy weight management
Source: Sci Rep. 2025 Nov 27;15:45510. doi: 10.1038/s41598-025-30028-y (PMC12749348; doi:10.1038/s41598-025-30028-y)
Supplement: Supplementary file 1 — Supplementary Information 1. [file 41598_2025_30028_MOESM1_ESM.docx]

**Figure S1. Calibration performance across different covariate selection scenarios**


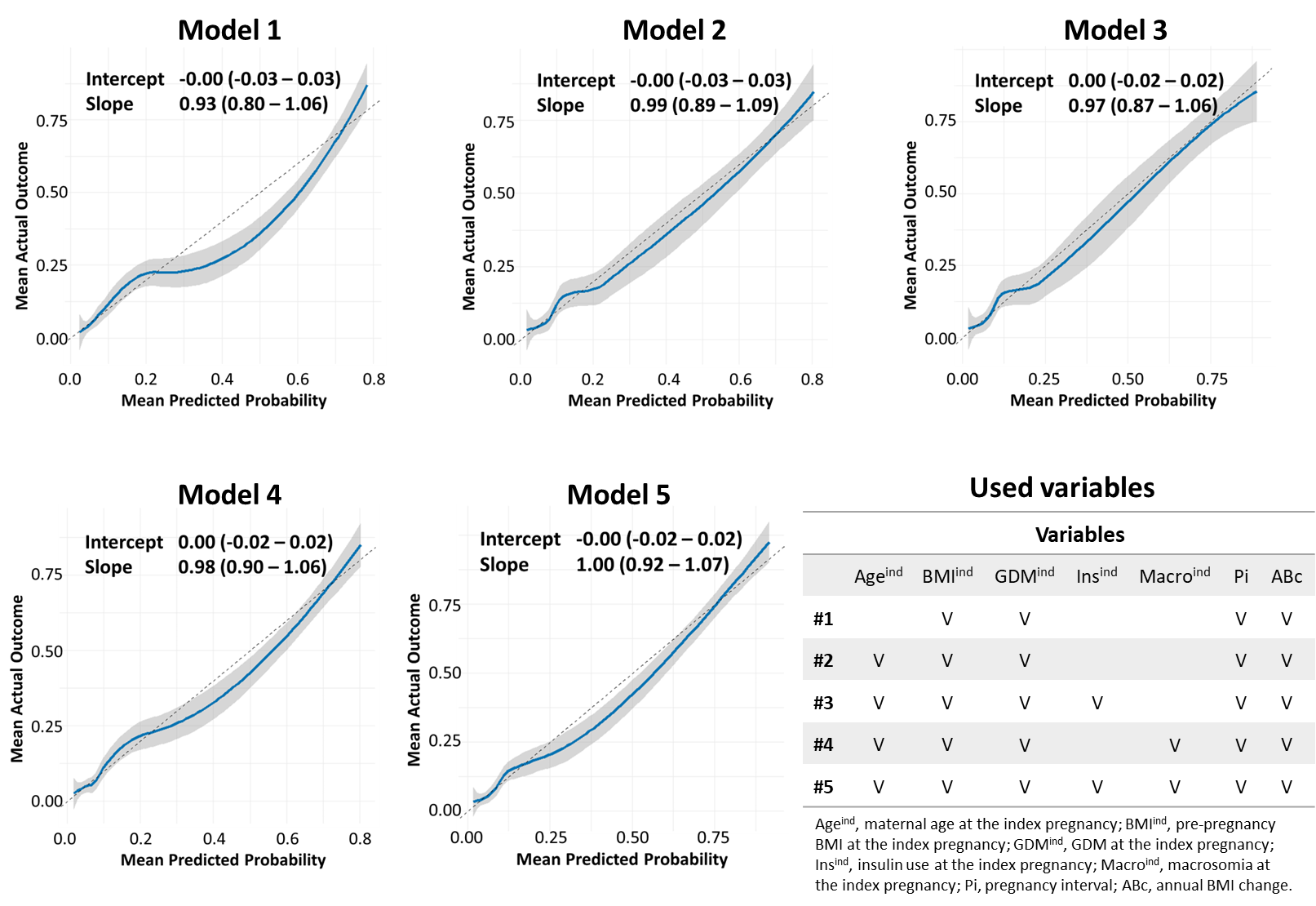


Calibration plots of logistic regression models for predicting GDM risk under five different covariate selection scenarios.

The x-axis represents predicted probabilities, and the y-axis shows observed event rates. The solid blue line indicates model calibration, and the dashed diagonal line denotes perfect calibration. Shaded areas represent 95% confidence intervals. Estimated intercepts and slopes (95% CIs) are displayed within each panel.

The table summarizes covariates included in each scenario.
