# Supplementary material for: Predicting gestational diabetes before conception for personalized interpregnancy weight management
Source: Sci Rep. 2025 Nov 27;15:45510. doi: 10.1038/s41598-025-30028-y (PMC12749348; doi:10.1038/s41598-025-30028-y)
Supplement: Supplementary file 2 — Supplementary Information 2. [file 41598_2025_30028_MOESM2_ESM.docx]

**Table S1. Comparison of the complete-case group (derivation cohort) and missing data group (excluded cohort)**

|  | Complete-case group (n = 1,640) | Missing data group  (n = 107) | Missing, n | *p*-value |
| --- | --- | --- | --- | --- |
| *Index pregnancy* |  |  |  |  |
| Age, years | 30.6 ± 4.8 | 29.1 ± 4.9 | 0 | 0.001* |
| Pre-pregnancy BMI, kg/m^2^ | 20.9 ± 3.4 | 20.9 ± 3.2 | 3 | 0.963 |
| Primiparity | 1,302 (79.4) | 84 (79.2) | 0 | 0.971 |
| GDM | 70 (4.3) | 1/8 (55.6) | 99 | 0.198 |
| GA at delivery, weeks | 39.1 ± 2.1 | 39.0 ± 1.7 | 0 | 0.645 |
| Birthweight, g | 2,966 ± 507 | 2,928 ± 489 | 0 | 0.450 |
| Macrosomia | 14 (0.9) | 1 (0.9) | 0 | 0.614 |
| *Interpregnancy period* |  |  |  |  |
| Pregnancy interval, years | 2.3 ± 0.9 | 1.8 ± 0.4 | 0 | <0.001* |
| Annual BMI change, kg/m^2^/year | 0.21 ± 0.80 | 0.29 ± 0.81 | 8 | 0.312 |
| *Subsequent pregnancy* |  |  |  |  |
| GDM | 156 (9.5) | 12 (11.2) | 0 | 0.563 |
| GA at delivery, weeks | 39.0 ± 1.6 | 39.0 ± 1.3 | 0 | 0.969 |
| Birthweight, g | 3,041 ± 436 | 2,987 ± 423 | 0 | 0.207 |
| Macrosomia | 19 (1.2) | 0 (0.0) | 0 | 0.625 |

Data are presented as mean ± standard deviation or n (%). BMI, body mass index; GDM, gestational diabetes mellitus; GA, gestational age.

* Statistically significant compared to the derivation cohort by t-test or chi-square test or Fisher’s exact test.
