# Supplementary material for: Predicting gestational diabetes before conception for personalized interpregnancy weight management
Source: Sci Rep. 2025 Nov 27;15:45510. doi: 10.1038/s41598-025-30028-y (PMC12749348; doi:10.1038/s41598-025-30028-y)
Supplement: Supplementary file 3 — Supplementary Information 3. [file 41598_2025_30028_MOESM3_ESM.docx]

**Table S2. Distribution of Predicted Probabilities Across Derivation, Temporal Validation, External Validation, and Overall Cohorts**

| Estimated Probability |  | Derivation | |  | Temporal- validation | |  | Geographical- validation | |  | Overall | |
| --- | --- | --- | --- | --- | --- | --- | --- | --- | --- | --- | --- | --- |
|  |  | n | Cumulative, % |  | n | Cumulative, % |  | n | Cumulative, % |  | n | Cumulative, % |
| 0-5% |  | 586 | 35.7 |  | 55 | 18.8 |  | 109 | 32.2 |  | 750 | 33.0 |
| 5-10% |  | 765 | 82.4 |  | 139 | 66.2 |  | 150 | 76.4 |  | 1,054 | 79.4 |
| 10-15% |  | 159 | 92.1 |  | 32 | 77.1 |  | 33 | 86.1 |  | 224 | 89.3 |
| 15-20% |  | 26 | 93.7 |  | 18 | 83.3 |  | 14 | 90.3 |  | 58 | 91.8 |
| 20-25% |  | 18 | 94.8 |  | 7 | 85.7 |  | 13 | 94.1 |  | 38 | 93.5 |
| 25-30% |  | 6 | 95.1 |  | 3 | 86.7 |  | 2 | 94.7 |  | 11 | 94.0 |
| 30-35% |  | 4 | 95.4 |  | 2 | 87.4 |  | 2 | 95.3 |  | 8 | 94.3 |
| 35-40% |  | 3 | 95.5 |  | 2 | 88.1 |  | 0 | 95.3 |  | 5 | 94.5 |
| 40-45% |  | 3 | 95.7 |  | 2 | 88.7 |  | 1 | 95.6 |  | 6 | 94.8 |
| 45-50% |  | 4 | 96.0 |  | 2 | 89.4 |  | 0 | 95.6 |  | 6 | 95.1 |
| 50-55% |  | 2 | 96.1 |  | 3 | 90.4 |  | 3 | 96.5 |  | 8 | 95.4 |
| 55-60% |  | 13 | 96.9 |  | 6 | 92.5 |  | 4 | 97.6 |  | 23 | 96.4 |
| 60-65% |  | 10 | 97.5 |  | 2 | 93.2 |  | 0 | 97.6 |  | 12 | 97.0 |
| 65-70% |  | 11 | 98.2 |  | 4 | 94.5 |  | 1 | 97.9 |  | 16 | 97.7 |
| 70-75% |  | 8 | 98.7 |  | 3 | 95.6 |  | 2 | 98.5 |  | 13 | 98.2 |
| 75-80% |  | 9 | 99.2 |  | 6 | 97.6 |  | 1 | 98.8 |  | 16 | 98.9 |
| 80-85% |  | 8 | 99.7 |  | 2 | 98.3 |  | 4 | 100.0 |  | 14 | 99.6 |
| 85-90% |  | 1 | 99.8 |  | 3 | 99.3 |  | 0 | 100.0 |  | 4 | 99.7 |
| 90-95% |  | 2 | 99.9 |  | 2 | 100.0 |  | 0 | 100.0 |  | 4 | 99.9 |
| 95-100% |  | 2 | 100.0 |  | 0 | 100.0 |  | 0 | 100.0 |  | 2 | 100.0 |
